# Tunable Hydrogen Evolution Reaction Property of Janus SWSe Monolayer Using Defect and Strain Engineering

**DOI:** 10.3390/molecules30071588

**Published:** 2025-04-02

**Authors:** Tian Chen, Lu Shen, Fuyuan Wang, Ping Jiang

**Affiliations:** 1 School of Mechanical Engineering, Wanjiang University of Technology, Maanshan 243031, China; 2 School of Civil Engineering, Wanjiang University of Technology, Maanshan 243031, China

**Keywords:** Janus structure, transition metal dichalcogenides, SWSe, external strain, first-principles calculation

## Abstract

Janus-structured transition metal dichalcogenides (TMDs) demonstrate remarkable electronic, optical, and catalytic characteristics owing to their distinctive asymmetric configurations. In this study, we comprehensively analyze the stability of Janus SWSe containing common vacancy defects through first-principles calculations. The findings indicate that the Gibbs free energy for the hydrogen evolution reaction (HER) is notably decreased to around 0.5 eV, which is lower compared with both pristine SWSe and traditional MoS_2_ monolayers. Importantly, the introduction of external strain further improves the HER efficiency of defect-modified Janus SWSe. This enhancement is linked to the adaptive relaxation of localized strain by unsaturated bonds in the defect area, leading to unique adjustable patterns. Our results clarify the fundamental mechanism driving the improved HER performance of SWSe via strain modulation, offering theoretical insights for designing effective HER catalysts using defective Janus TMDs.

## 1. Introduction

The breakthrough in isolating graphene in 2004 sparked widespread research, emphasizing its remarkable electronic, optical, thermal, and mechanical characteristics [1,2]. Nonetheless, its semimetallic behavior, marked by the absence of a bandgap, restricts its application in nanoscale devices [3,4]. As a result, transition metal dichalcogenides (TMDs) have gained attention as a novel category of two-dimensional semiconductors, offering superior stability and ease of fabrication [5,6,7]. These materials not only share a hexagonal lattice structure akin to graphene, but also feature a measurable bandgap [8]. Notable instances like MoS_2_ and WS_2_ exhibit outstanding electronic and optical traits, positioning them as strong contenders for use in photocatalysis, solar energy conversion, and optoelectronics [9]. Specifically, single-layer MoS_2_ has shown electron mobility reaching ~200 cm^2^/V·s and a direct bandgap of around 1.8 eV, underscoring its suitability for energy-efficient transistors [10].

In contrast with traditional TMDs, Janus SWSe is created by substituting a sulfur (S) atomic layer with selenium (Se) atoms [11], resulting in the loss of mirror symmetry and the creation of an inherent electric field [12]. This structural arrangement gives rise to distinct electronic, optical, and dipole characteristics [13]. Within the SWSe monolayer, an electric field forms from the Se layer to the S layer, leading to electron enrichment in the Se layer and hole accumulation in the S layer, thereby lowering the exciton binding energy [14]. Additionally, SWSe monolayers demonstrate a peak electron mobility of around 2443.2 cm^2^/V·s under ideal stacking conditions, highlighting their promising use in electronic devices [15]. Moreover, the built-in vertical electric fields improve light absorption and, when incorporated into van der Waals (vdW) heterostructures, can reduce rapid electron−hole recombination rates, positioning them as effective photocatalysts for water splitting [16].

Although previous studies indicate that Janus SWSe monolayers possess a superior catalytic potential compared with traditional TMDs [17,18,19], the synthesis of these materials often results in various vacancy-related imperfections [20,21]. These defects can substantially modify electronic behaviors and structural attributes, impacting both catalytic efficiency and mechanical properties [22]. Research has demonstrated that S vacancies are frequently observed in MoS_2_, whereas W vacancies are more common in WSe_2_ [23]. Additionally, localized states tend to form around these vacancies, altering electronic configurations and potentially improving the catalytic activity. Computational studies reveal that S, Se, Mo, and Se−S vacancies in SWSe exhibit low formation energies, indicating their ease of generation [24,25]. The presence of Mo vacancies can notably adjust the bandgap while diminishing the intrinsic magnetic moments [26]. On the other hand, S and Se vacancies can elevate and reduce electrostatic potential differences across dipole moments, respectively, shifting electron cloud distributions around Mo atoms [26]. Moreover, vacancies create numerous adsorption sites for catalytic processes, with electronic modifications influencing catalytic outcomes [27]. The introduction of defects also leads to localized strain distributions, which can optimize adsorption sites by manipulating electron clouds near vacancies [23]. Recent investigations highlight strain engineering as a powerful approach for adjusting the catalytic properties of 2D materials [28]. This is especially relevant for Janus structures, whose inherent asymmetry and unique electric fields make them highly responsive to external forces, positioning strain engineering as a vital strategy for enhancing hydrogen evolution reaction (HER) efficiency. Consequently, defect and strain engineering are likely to be pivotal in modulating the catalytic performance in Janus TMDs.

In this research, we methodically created SWSe monolayers featuring six distinct vacancy-related imperfections and assessed their structural integrity through first-principles computational methods. Following this, we explored how these intentionally altered SWSe layers perform as catalysts for the hydrogen evolution reaction (HER), paying close attention to how different vacancy types influence their efficiency. Moreover, by introducing external strain to these imperfect Janus structures, we observed a notable improvement in their catalytic capabilities for HER.

## 2. Results and Discussion

**Figure 1:**
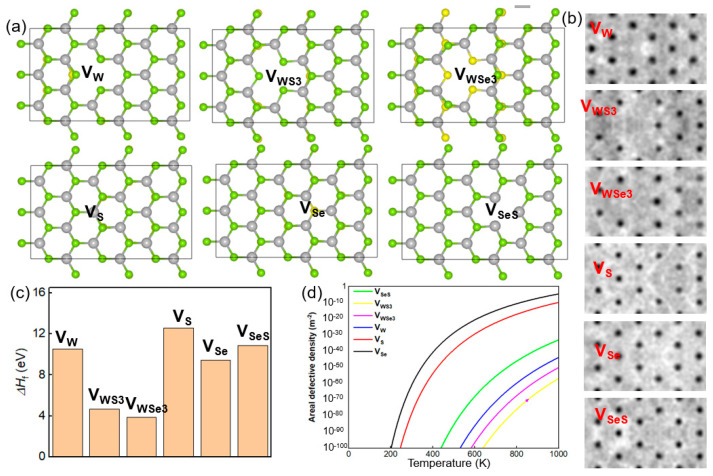
(**a**) The atomic structures and (**b**) corresponding STM images (bottom) for six distinct types of Janus SWSe monolayers featuring vacancy defects, identified as V_W_, V_S_, V_Se_, V_SeS_, V_WS3_, and V_WSe3_. The yellow, green, and blue spheres denote S, Se, and W atoms, respectively. (**c**) The computed formation energy and (**d**) areal defect density for these defective Janus SWSe monolayers.

To model the hydrogen adsorption on a SWSe monolayer, a 3 × 6 × 1 supercell was utilized. Following comprehensive relaxation, the pristine SWSe monolayer displayed lattice parameters of 16.77 Å on the *x*-axis and 9.68 Å on the y-axis, as depicted in Figure 1a. The Mo–Se and Mo–S bond lengths were determined to be 2.530 Å and 2.412 Å, respectively, aligning with the previously reported data [29]. Subsequently, six distinct vacancy defect configurations were created and designated as V_W_, V_S_, V_Se_, V_SeS_, V_WS3_, and V_WSe3_, as illustrated in Figure 1a. The V_W_, V_S_, and V_Se_ configurations were achieved by eliminating one W, S, and Se atom, respectively, while V_SeS_ involved removing one S and one Se atom from mirror-symmetric layers. Additionally, V_WS3_ was formed by removing one W atom and three neighboring S atoms, and V_WSe3_ by removing one W atom and three adjacent Se atoms. The simulated STM images of these defective SWSe monolayers are also presented in Figure 1a, offering theoretical insights for experimental investigations.

Following relaxation, the distances between W–S and W–Se bonds surrounding the defects showed notable alterations. In the V_W_ structure, W–S bonds adjacent to the defect were significantly reduced, whereas W–Se bonds experienced a minor extension. Conversely, for the V_S_, V_Se_, and V_SeS_ structures, both W–S and W–Se bonds close to the defects underwent slight contraction. Additionally, the V_WS3_ and V_WSe3_ structures demonstrated marked reductions in W–Se and W–S bond lengths near the defects, respectively. The formation energy (Δ*H*_f_) for defective SWSe monolayers was determined as follows:

Δ*H*_f_ = *H*_a_ − *H*_b_ − *H*_c_. (1)

Here, *H*_a_ represents the total energy of the intact SWSe monolayer, *H*_b_ denotes the total energy of the SWSe monolayer with defects, and *H*_c_ signifies the total energy of the atoms that have been removed. The formation energies for defects such as V_W_, V_S_, V_Se_, V_SeS_, V_WS3_, and V_WSe3_ were determined to be 10.54 eV, 12.59 eV, 10.89 eV, 4.67 eV, 3.86 eV, and 9.45 eV, respectively, as illustrated in Figure 1b. It is evident that the formation energies for V_S_ and V_Se_ defects were comparatively lower, whereas those associated with W atoms were significantly higher. This suggests that while the synthesis of a pristine Janus SWSe structure is achievable, the creation of atomic vacancies might necessitate the use of high-energy particle bombardment techniques.

To investigate the structural stability of the defective SWSe monolayer in greater depth, the surface defect concentration was determined through the following calculation:

(2) $$n=n_{0}\exp(\frac{-E}{KT}).$$

*n*_0_ denotes the actual atomic density, *E* stands for the energy required for defect formation, *K* is the Boltzmann constant, and *T* refers to the temperature during synthesis. The calculated atomic densities for V_W_, V_S_, V_Se_, V_SeS_, V_WS3_, and V_WSe3_ are 3.262 × 10^19^ m^−2^, 3.078 × 10^19^ m^−2^, 3.078 × 10^19^ m^−2^, 3.262 × 10^19^ m^−2^, 3.262 × 10^19^ m^−2^, and 3.201 × 10^19^ m^−2^, respectively, as illustrated in Figure 1c. These findings suggest that even under elevated temperatures, the densities of these defects remain relatively low, highlighting the essential role of particle bombardment in generating such defects.

**Figure 2:**
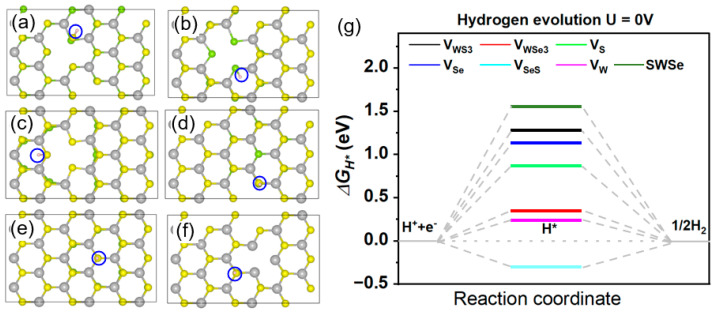
The optimal binding locations (**a**–**f**) marked by blue circel and associated Gibbs free energy values (**g**) for six distinct defect configurations in the SWSe monolayer. H* demonstrates the stable configuration of intermediates.

The Gibbs free energy associated with hydrogen adsorption in the Volmer reaction was examined for six distinct defective SWSe configurations. Following comprehensive relaxation and energy self-consistent computations of the hydrogen-adsorbed systems, the most stable hydrogen adsorption sites were determined for each defective SWSe monolayer, as illustrated in Figure 2a–f. In the case of V_W_ and V_WS3_ defects, hydrogen atoms exhibited a tendency to form bonds with selenium atoms located at the edges of atomic vacancies. Conversely, for V_WSe3_, V_S_, V_Se_, and V_SeS_ structures, hydrogen atoms preferentially adsorbed onto sulfur atom sites at the defect edges. The computed Gibbs free energies of hydrogen adsorption for the defective SWSe monolayers are depicted in Figure 2g. It is evident that these values were nearer to zero in comparison with those of pristine SWSe, suggesting that vacancy defects can markedly improve the hydrogen evolution reaction (HER) performance of the SWSe monolayer. For V_S_, V_Se_, and V_SeS_ configurations, despite their enhanced catalytic performance relative to the perfect structure, the Gibbs free energy still surpassed 1.0 eV, implying that the interaction between hydrogen atoms and the material was insufficient to achieve an optimal catalytic performance. In contrast, for the V_W_, V_WS3_, and V_WSe3_ configurations, the Gibbs free energy of hydrogen adsorption was relatively closer to zero, indicating that the energy investment required to create these defects and activate catalytic sites was warranted. The positive Gibbs free energy observed for V_WS3_ and V_WSe3_ structures suggests weaker Volmer reactions, which are less favorable for hydrogen adsorption, whereas the negative Gibbs free energy for the V_W_ structure indicates a stronger Volmer reaction, as demonstrated in Figure 2g.

**Figure 3:**
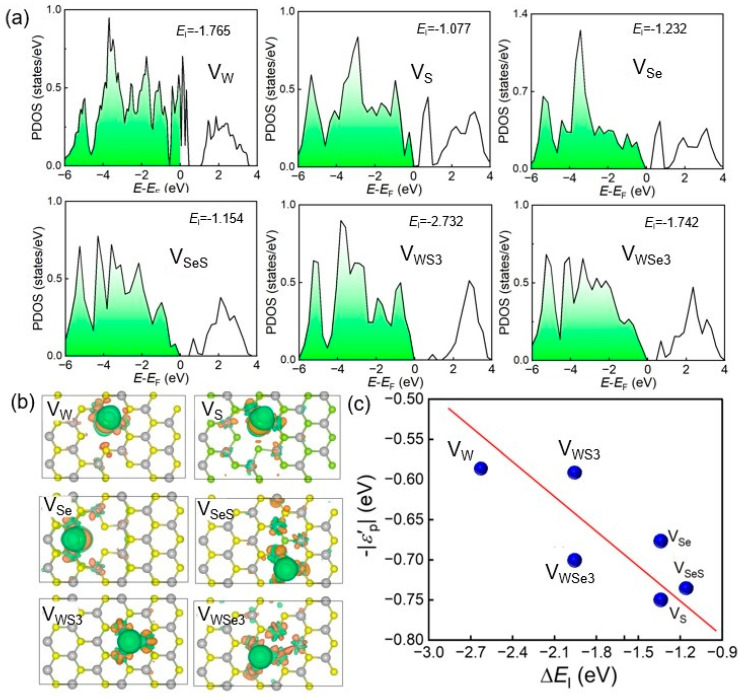
The anticipated density of states (**a**), variation in charge density (**b**), and estimated band center (**c**) for SWSe monolayers with defects. The Fermi level and the isosurface threshold for charge variation are established at 0 eV and 10^−3^|e|, respectively. The accumulation and depletion of electrons are indicated by yellow and blue markers, respectively. The red line the fitted band center of WSSe with defects.

The electronic configuration of the SWSe monolayer significantly influenced its catalytic performance in the hydrogen evolution reaction (HER). Analyzing the electronic properties of defective SWSe monolayers is vital for comprehending their catalytic behavior. Figure 3 displays the density of states (DOS) for these defective systems, emphasizing the importance of the p orbitals of S and Se atoms in the formation of H–S and H–Se bonds, as depicted in Figure 3a. To delve deeper into the electronic characteristics of these six defect types, differential charge density diagrams for each are provided in Figure 3b. These diagrams reveal that H atoms in all six configurations were enveloped by areas rich in electrons (highlighted in blue). Nonetheless, with the exception of the V_MoS3_ structure, the other defect types also displayed regions lacking electrons (marked in cyan) around the H atoms. In the charge density difference diagram in Figure 3b, the H atom in the V_MoS3_ structure acquired electrons, whereas the H atoms in the V_Se_, V_S_, V_Mo_, V_SeS_, and V_MoSe3_ structures lost electrons. The charge transfer of the H atoms in these six defective systems was also assessed using the Bader method [30]. These findings support our hypotheses, with the charge transfer values for H atoms in the V_Mo_, V_MoS3_, V_MoSe3_, V_S_, V_Se_, and V_SeS_ configurations being −0.051, 0.084, −0.034, −0.070, −0.055, and −0.093, respectively. The p-projected orbitals and the projection band center for these systems are illustrated in Figure 3c. The projection band center of the SWSe monolayer was calculated as follows:

(3) $$\varepsilon =\frac{\int_{-\infty }^{\infty }x\rho (x)dx}{\int_{-\infty }^{\infty }\rho (x)dx}.$$

The Δ*E*_I_ values exhibited a roughly linear trend with respect to −|*ε*_p_|. While a distinct linear relationship between Δ*E*_I_ and −|*ε*_p_ | was not evident across the full spectrum, limiting the integration range to −3 to 0 eV uncovered a slight linear correlation. When the calculation range for the p-band center was further narrowed to −1 to 0 eV, a robust linear relationship emerged for all structures except V_WS3_. This indicates that the creation of Se–H and S–H bonds during catalysis was mainly influenced by the p-orbitals of Se or S atoms at the active sites. Additionally, it underscores the importance of states near the Fermi level in the p orbitals, with adsorption strength increasing as −|*ε*_p_| neared the Fermi level. The anomaly in the V_WS3_ data point was due to the active site being on Se atoms rather than S atoms, meaning the computed band center corresponded to Se rather than S. It is worth noting that the defect controlled catalytic performance of TMDs has also been widely validated in experiments. For example, MoS_2_/FeS_2_/C was prepared with abundant defects and a hollow structure, and the defected interface could relieve the lattice stress and volume change sequentially so that the anode showed an excellent capacity of 613.1 mA h g^−1^ at 0.5 A g^−1^ and 306.1 mA h g^−1^ at 20 A g^−1^, which was also confirmed by DFT simulations [31]. The 2D MoS_2(1−*x*)_Se_2*x*_ nanoflakes were obtained and the introduction of Se continuously tuned the d band electronic structure of Mo; thus, the hydrogen adsorption free energy and electrocatalytic activity were improved [32].

**Figure 4:**
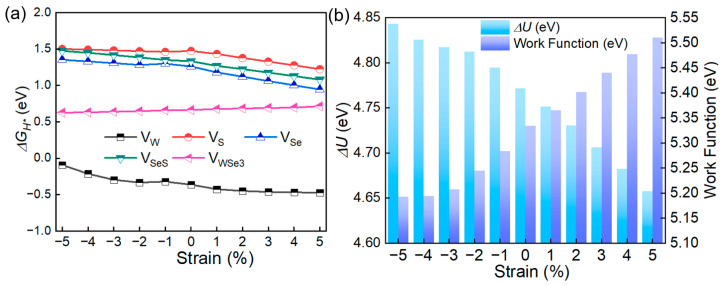
(**a**) The influence of strain on the Gibbs free energy for the SWSe monolayer with defects. (**b**) Voltage drop and work function of the V_W_ structure under applied strain.

Recent research has shown that strain engineering serves as a powerful approach for altering structural attributes, thus adjusting the chemical and electronic properties of two-dimensional (2D) materials [33,34,35,36]. Particularly, the catalytic efficiency of 2D materials can be substantially improved through the application of external strain [37]. Further, the Janus SWSe and SMoSe can withstand large external strains, and their horizontal heterojunctions even exhibit super flexible characteristics due to their special structure [38]. Thus, external biaxial strain (*ε*) ranging from −5.0% to 5.0% was applied to different defective SWSe configurations to explore their hydrogen evolution reaction (HER) capabilities, as illustrated in Figure 4a. The Gibbs free energies associated with hydrogen adsorption for the V_W_, V_SeS_, V_S_, and V_Se_ defective structures showed a direct relationship with strain, decreasing as the structures shift from compression to tension. In contrast, the V_WS3_ and V_WSe3_ configurations exhibit an inverse trend. Additionally, the HER catalytic performance of the V_W_, V_SeS_, V_S_, and V_Se_ defective SWSe is highly responsive to strain, while the V_WS3_ and V_WSe3_ structures demonstrated a relative insensitivity to biaxial strain. It is important to highlight that the V_WS3_ structure becomes less stable under a strain of −2.0%. Among the V_W_, V_SeS_, V_S_, and V_Se_ systems, the V_W_ structure displayed the most pronounced mechanical tuning effect, with the hydrogen adsorption Gibbs free energy nearing zero at ε = −5.0%.

To delve deeper into how external strain influences the electronic configuration of defective SWSe, the intrinsic electric field potential and work function of these systems were analyzed, as depicted in Figure 4b. The V_W_ setup exhibited a reduced potential drop at the interface when subjected to strain, whereas the work function increased, suggesting improved stability for hydrogen attachment [39]. Despite the external strain diminishing the potential drop at the interface, it still surpassed that of many documented TMD substances [30,31]. This significant potential drop in flawed Janus SWSe is pivotal for the segregation of photo-induced electrons and holes [40].

**Figure 5:**
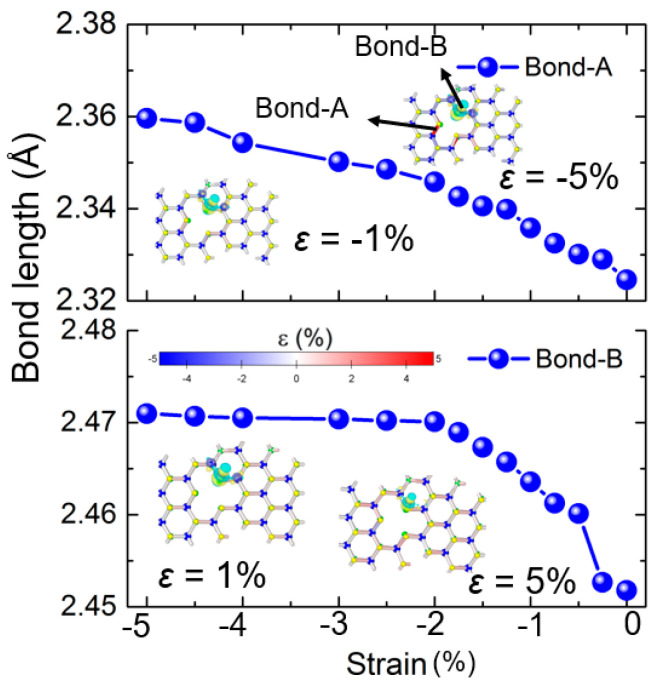
The bond stress and the variation in charge distribution of the defective SWSe monolayer featuring the V_W_ arrangement under varying external stresses. The inset is the correlation between the bond stress and external stress for Bond A and Bond B.

Further analysis was conducted on the bond properties within hydrogen-adsorbed defective SWSe systems to understand how strained bonds affect the hydrogen evolution reaction (HER). Figure 5 displays the bond strain of a defective V_W_ configuration monolayer under varying external strains from −0.01 to −0.05 and 0.01 to 0.05. It is clear that as external strain intensified, the general bond length extended, shown by the prevalence of blue (compressed) and red (extended) bonds. However, bond strains in the defective area varied with external strain, as these bonds were in a suspended state and more prone to the influence of adsorbed hydrogen. For instance, the inset in the Figure 5 shows that the bond length of Bond A, highlighted in Figure 5, lengthened with external compressive strain. Remarkably, even Bond B, which was far from the hydrogen, stretched under compressive strain, due to the compressive-induced bulging out-of-plane of the Janus SWSe monolayer. Moreover, as the overall bond strain of the system increased during the catalyst’s stretching phase, the charge density difference in the hydrogen-adsorbed SWSe system could also be amplified (Figure 5). This insight is vital for enhancing the HER efficiency of Janus SWSe through external strain.

More importantly, the experimental work also confirmed the effectiveness of stress regulation on the catalytic properties of MTDs. The strain tunable electronic and catalyst performances of the WS_2_ monolayer was investigated, using the synthesized chemical vapor deposition (CVD)-grown method [41]. Then, the catalyst ability of the WS_2_ monolayer can be modulated by the band structure under external strain [42]. Further, chemically exfoliated WS_2_ serves as a highly efficient catalyst for hydrogen evolution, exhibiting very low overpotentials in experiments [43]. The electronic structure of atomically thin MoS_2_ on flexible substrates can be continuously tuned under uniaxial tensile strain. Absorption and photoluminescence spectroscopy revealed a redshift of ~70 meV/% strain for direct bandgap transitions and a 1.6× larger shift for indirect transitions. Through photoluminescence imaging, the strain-mediated decrease in the direct bandgap and the directional flow of photogenerated excitons toward areas of increased strain [44].

## 3. Computational Methods

All of the calculations were conducted using the Vienna Ab initio Simulation Package (VASP) version 6.3.2, which is grounded in density functional theory (DFT) [45,46]. The generalized gradient approximation (GGA) was applied, specifically employing the Perdew–Burke–Ernzerhof (PBE) functional [47,48,49]. Wave functions were expanded using a plane wave basis set, with a cutoff energy set at 450 eV [50,51]. Sampling of the Brillouin zones was carried out using k-point meshes that were equally spaced and sufficiently dense. For structural relaxation, a Monkhorst−Pack k-point grid of 7 × 7 × 1 was used, while an 11 × 11 × 1 grid was utilized for electronic structure computations. To avoid interactions between neighboring slabs, a vacuum layer of 20 Å was introduced. The convergence thresholds for energy and force were established at 10−^5^ eV and 0.01 eV/Å, respectively. Additionally, the Gibbs free energy for hydrogen adsorption under standard conditions was determined as follows:

(4) $$∆G_{H^{*}}=∆E_{H^{*}}+∆E_{ZEP}-T∆S_{H^{*}}$$

where $∆G_{H^{*}}$, $∆E_{H^{*}}$, $∆E_{ZEP}$, and $∆S_{H^{*}}$ denotes the Gibbs free energy associated with hydrogen adsorption, the energy required for hydrogen adsorption, the correction for zero-point energy, and the change in entropy between adsorbed hydrogen and hydrogen in the gaseous state, respectively. The energy related to hydrogen adsorption is expressed as follows:

(5) $$∆E_{H^{*}}=E_{Cat-H}-E_{Cat}-\frac{1}{2}E_{H_{2}}$$

where $E_{Cat-H},E_{Cat}$ and $E_{H_{2}}$ represent the overall energy levels of the catalyst with hydrogen adsorption, the unmodified catalyst, and the standalone H_2_ molecule, respectively. Additionally, the correction for zero-point energy is computed by analyzing the vibrational frequencies of adsorbed hydrogen $\sum \hbar \omega_{i}(H^{*})$ and the H_2_ molecule $\hbar \omega \left(H_{2}\right)$ as [52]:

(6) $$∆E_{ZEP}=\sum \hbar \omega_{i}(H^{*})-\frac{1}{2}\hbar \omega \left(H_{2}\right)$$

Lastly, the change in entropy ($∆S_{H^{*}}$) between the hydrogen in the adsorbed state and the hydrogen in the gaseous phase H_2_ is defined as follows:

(7) $$∆S_{H^{*}}=S_{H^{*}}-\frac{1}{2}S_{H_{2}}$$

Typically, $S_{H^{*}}$ and $S_{H_{2}}$ denote the entropy values for adsorbed hydrogen and gaseous H_2_, respectively.

In addition, external stress is introduced by adjusting the lattice parameters of the system, varying from −0.05 to 0.05. Positive values represent tensile forces, while negative values denote compressive forces. The bond strain (*ε*_b_) for the system under investigation is determined through the following method [53]:

(8) $$\varepsilon_{b}=\frac{l-l_{0}}{l_{0}}$$

In this context, *l* and *l*_0_ denote the initial and deformed bond lengths within the SWSe monolayer, respectively.

## 4. Conclusions

To encapsulate, Janus SWSe showcases distinct electronic characteristics that make it an excellent prospect for hydrogen evolution reaction (HER) catalysis. The introduction of vacancy defects further boosts its HER efficiency. Utilizing first-principles computations, we determined the stable configurations of vacancy-defective Janus SWSe and noted a considerable decrease in Gibbs free energy for HER relative to the pristine SWSe monolayer, as elucidated by p-band center theory. Additionally, the imposition of external biaxial strain effectively modulates the HER activity of the vacancy-defective Janus SWSe monolayer. The dangling bonds at defect sites spontaneously adapt the structure to mitigate localized strain, thereby improving the HER performance. This enhancement is also associated with alterations in the built-in electric field and work function. Our results indicate that the Janus SWSe monolayer, with strategically designed structural defects, holds significant promise as a highly efficient HER catalyst.

## Data Availability

The data presented in this study are available upon request from the corresponding author.

## References

[1] Geim A.K., Novoselov K.S. (2007). The rise of graphene. Nat. Mater..

[2] Ren K., Liu J.Z., Palummo M., Sun M. (2024). Theoretical study of two-dimensional materials for photocatalysis and photovoltaics. Front. Chem..

[3] Ren K., Chen Y., Qin H., Feng W., Zhang G. (2022). Graphene/biphenylene heterostructure: Interfacial thermal conduction and thermal rectification. Appl. Phys. Lett..

[4] Ren K., Wang K., Luo Y., Sun M., Altalhi T., Yakobson B.I., Zhang G. (2025). Ultralow Frequency Interlayer Mode from Suppressed van der Waals Coupling in Polar Janus SMoSe/SWSe Heterostructure. Mater. Today Phys..

[5] Zhang H., Chhowalla M., Liu Z. (2018). 2D nanomaterials: Graphene and transition metal dichalcogenides. Chem. Soc. Rev..

[6] Shu H., Wang F., Ren K., Guo J. (2025). Strain-tunable optoelectronic and photocatalytic properties of 2D GaN/MoSi_2_P_4_ heterobilayers: Potential optoelectronic/photocatalytic materials. Nanoscale.

[7] Gui J.-X., Cheng Y., Ren K., Liu Z.-P., Zhu Z., Xue Z.-Y., Zhu Y., Wang R.-H., Pei G., Sui J. (2025). Development of Ternary Hydrogel Electrolytes for Superior Gel Thermocells: Exceptional Anti-Drying, Anti-Freezing, and Mechanical Robustness. Adv. Mater..

[8] Ren K., Sun M., Luo Y., Wang S., Yu J., Tang W. (2019). First-principle study of electronic and optical properties of two-dimensional materials-based heterostructures based on transition metal dichalcogenides and boron phosphide. Appl. Surf. Sci..

[9] Luo Y., Ren K., Wang S., Chou J.-P., Yu J., Sun Z., Sun M. (2019). First-Principles Study on Transition-Metal Dichalcogenide/BSe van der Waals Heterostructures: A Promising Water-Splitting Photocatalyst. J. Phys. Chem. C.

[10] Cai Y., Zhang G., Zhang Y.W. (2014). Polarity-reversed robust carrier mobility in monolayer MoS_2_ nanoribbons. J. Am. Chem. Soc..

[11] Dong L., Lou J., Shenoy V.B. (2017). Large In-Plane and Vertical Piezoelectricity in Janus Transition Metal Dichalchogenides. ACS Nano.

[12] Lu A.Y., Zhu H., Xiao J., Chuu C.P., Han Y., Chiu M.H., Cheng C.C., Yang C.W., Wei K.H., Yang Y. (2017). Janus monolayers of transition metal dichalcogenides. Nat. Nanotechnol..

[13] Zhang K., Guo Y., Ji Q., Lu A.Y., Su C., Wang H., Puretzky A.A., Geohegan D.B., Qian X., Fang S. (2020). Enhancement of van der Waals Interlayer Coupling through Polar Janus SWSe. J. Am. Chem. Soc..

[14] Jin C., Tang X., Tan X., Smith S.C., Dai Y., Kou L. (2019). A Janus SWSe monolayer: A superior and strain-sensitive gas sensing material. J. Mater. Chem. A.

[15] Ren K., Wang S., Luo Y., Chou J.-P., Yu J., Tang W., Sun M. (2020). High-efficiency photocatalyst for water splitting: A Janus SWSe/XN (X = Ga, Al) van der Waals heterostructure. J. Phys. Phys. D Appl. Phys..

[16] Li F., Wei W., Zhao P., Huang B., Dai Y. (2017). Electronic and Optical Properties of Pristine and Vertical and Lateral Heterostructures of Janus SWSe and WSSe. J. Phys. Chem. Lett..

[17] Zhang C., Ren K., Wang S., Luo Y., Tang W., Sun M. (2023). Recent progress on two-dimensional van der Waals heterostructures for photocatalytic water splitting: A selective review. J. Phys. Phys. D Appl. Phys..

[18] Peng R., Ma Y., Zhang S., Huang B., Dai Y. (2018). Valley Polarization in Janus Single-Layer SWSe via Magnetic Doping. J. Phys. Chem. Lett..

[19] Xia C., Xiong W., Du J., Wang T., Peng Y., Li J. (2018). Universality of electronic characteristics and photocatalyst applications in the two-dimensional Janus transition metal dichalcogenides. Phys. Rev. B.

[20] Wu X., Wang X., Li H., Zeng Z., Zheng B., Zhang D., Li F., Zhu X., Jiang Y., Pan A. (2019). Vapor growth of WSe2/WS2 heterostructures with stacking dependent optical properties. Nano Res..

[21] Zhao L., Huang L., Wang K., Mu W., Wu Q., Ma Z., Ren K. (2024). Mechanical and Lattice Thermal Properties of Si-Ge Lateral Heterostructures. Molecules.

[22] Ren K., Shu H., Huo W., Cui Z., Xu Y. (2022). Tuning electronic, magnetic and catalytic behaviors of biphenylene network by atomic doping. Nanotechnology.

[23] Hu Z., Wu Z., Han C., He J., Ni Z., Chen W. (2018). Two-dimensional transition metal dichalcogenides: Interface and defect engineering. Chem. Soc. Rev..

[24] Mehdipour H., Kratzer P. (2022). Structural defects in a Janus SWSe monolayer: A density functional theory study. Phys. Rev. B.

[25] Wang Y., Chen R., Luo X., Liang Q., Wang Y., Xie Q. (2022). First-principles calculations on Janus SWSe/graphene van der Waals heterostructures: Implications for electronic devices. ACS Appl. Nano Mater..

[26] Shi W., Li G., Wang Z. (2019). Triggering Catalytic Active Sites for Hydrogen Evolution Reaction by Intrinsic Defects in Janus Monolayer SWSe. J. Phys. Chem. C.

[27] Pu M., Guo Y., Guo W. (2021). Wrinkle facilitated hydrogen evolution reaction of vacancy-defected transition metal dichalcogenide monolayers. Nanoscale.

[28] Ren K., Shu H., Huo W., Cui Z., Yu J., Xu Y. (2021). Mechanical, electronic and optical properties of a novel B2P6 monolayer: Ultrahigh carrier mobility and strong optical absorption. Phys. Chem. Chem. Phys..

[29] Sanville E., Kenny S.D., Smith R., Henkelman G. (2007). Improved grid-based algorithm for Bader charge allocation. J. Comput. Chem..

[30] Ren K., Tang W., Sun M., Cai Y., Cheng Y., Zhang G. (2020). A direct Z-scheme PtS_2_/arsenene van der Waals heterostructure with high photocatalytic water splitting efficiency. Nanoscale.

[31] Cui Z., Ren K., Zhao Y., Wang X., Shu H., Yu J., Tang W., Sun M. (2019). Electronic and optical properties of van der Waals heterostructures of g-GaN and transition metal dichalcogenides. Appl. Surf. Sci..

[32] Ma L., Zhou X., Sun J., Zhang P., Hou B., Zhang S., Shang N., Song J., Ye H., Shao H. (2023). Synergy mechanism of defect engineering in MoS_2_/FeS_2_/C heterostructure for high-performance sodium-ion battery. J. Energy Chem..

[33] Gong Q., Cheng L., Liu C., Zhang M., Feng Q., Ye H., Zeng M., Xie L., Liu Z., Li Y. (2015). Ultrathin MoS_2(1–_*_x_*_)_Se_2*x*_ alloy nanoflakes for electrocatalytic hydrogen evolution reaction. ACS Catal..

[34] Shi H., Pan H., Zhang Y.-W., Yakobson B.I. (2013). Quasiparticle band structures and optical properties of strained monolayer MoS_2_and WS_2_. Phys. Rev. B.

[35] Mao X., Qin Z., Ge S., Rong C., Zhang B., Xuan F. (2023). Strain engineering of electrocatalysts for hydrogen evolution reaction. Mater. Horiz..

[36] Pu M., Guo Y., Guo W. (2022). Strain-mediated oxygen evolution reaction on magnetic two-dimensional monolayers. Nanoscale Horiz.

[37] Ren K., Luo Y., Wang S., Chou J.-P., Yu J., Tang W., Sun M. (2019). A van der Waals Heterostructure Based on Graphene-like Gallium Nitride and Boron Selenide: A High-Efficiency Photocatalyst for Water Splitting. ACS Omega.

[38] Ren K., Zhang G., Zhang L., Qin H., Zhang G. (2023). Ultraflexible two-dimensional Janus heterostructure superlattice: A novel intrinsic wrinkled structure. Nanoscale.

[39] Kulish V.V., Malyi O.I., Persson C., Wu P. (2015). Adsorption of metal adatoms on single-layer phosphorene. Phys. Chem. Chem. Phys..

[40] Ren K., Wang K., Cheng Y., Tang W., Zhang G. (2020). Two-dimensional heterostructures for photocatalytic water splitting: A review of recent progress. Nano Futures.

[41] Wang Y., Cong C., Yang W., Shang J., Peimyoo N., Chen Y., Kang J., Wang J., Huang W., Yu T. (2015). Strain-induced direct–indirect bandgap transition and phonon modulation in monolayer WS_2_. Nano Res..

[42] Voiry D., Yamaguchi H., Li J., Silva R., Alves D.C., Fujita T., Chen M., Asefa T., Shenoy V.B., Eda G. (2013). Enhanced catalytic activity in strained chemically exfoliated WS_2_ nanosheets for hydrogen evolution. Nat. Mater..

[43] He K., Poole C., Mak K.F., Shan J. (2013). Experimental demonstration of continuous electronic structure tuning via strain in atomically thin MoS_2_. Nano Lett..

[44] Castellanos-Gomez A., Roldan R., Cappelluti E., Buscema M., Guinea F., van der Zant H.S., Steele G.A. (2013). Local strain engineering in atomically thin MoS_2_. Nano Lett..

[45] Capelle K. (2006). A bird’s-eye view of density-functional theory. Braz. J. Phys..

[46] Grest G., Nagel S., Rahman A., Witten T.A. (1981). Density of states and the velocity autocorrelation function derived from quench studies. J. Chem. Phys..

[47] Kresse G., Furthmüller J. (1996). Efficiency of ab-initio total energy calculations for metals and semiconductors using a plane-wave basis set. Comp. Mater. Sci..

[48] Perdew J.P., Burke K., Ernzerhof M. (1996). Generalized gradient approximation made simple. Phys. Rev. Lett..

[49] Grimme S. (2006). Semiempirical GGA-type density functional constructed with a long-range dispersion correction. J. Comput. Chem..

[50] Kresse G., Joubert D. (1999). From ultrasoft pseudopotentials to the projector augmented-wave method. Phys. Rev. B.

[51] Blöchl P.E. (1994). Projector augmented-wave method. Phys. Rev. B.

[52] Zang Y., Wu Q., Du W., Dai Y., Huang B., Ma Y. (2021). Activating electrocatalytic hydrogen evolution performance of two-dimensional MSi_2_N_4_ (M=Mo, W): A theoretical prediction. Phys. Rev. Mater..

[53] Ren K., Qin H., Liu H., Chen Y., Liu X., Zhang G. (2022). Manipulating Interfacial Thermal Conduction of 2D Janus Heterostructure via a Thermo-Mechanical Coupling. Adv. Funct. Mater..

